# Detection of *Phenuiviridae, Chuviridae* Members, and a Novel *Quaranjavirus* in Hard Ticks From Danube Delta

**DOI:** 10.3389/fvets.2022.863814

**Published:** 2022-04-13

**Authors:** Bianca Elena Bratuleanu, Sarah Temmam, Sandie Munier, Delphine Chrétien, Thomas Bigot, Sylvie van der Werf, Gheorghe Savuta, Marc Eloit

**Affiliations:** ^1^Pathogen Discovery Laboratory, Institut Pasteur, Paris, France; ^2^Regional Center of Advanced Research for Emerging Diseases, Zoonoses and Food Safety (ROVETEMERG), “Ion Ionescu de la Brad”, University of Life Sciences, Iasi, Romania; ^3^Institut Pasteur, OIE Collaborating Centre for Detection and Identification in Humans of Emerging Animal Pathogens, Paris, France; ^4^Institut Pasteur, Molecular Genetics of RNA Viruses Unit, CNRS UMR 3569, Université de Paris, Paris, France; ^5^Institut Pasteur, National Reference Center for Respiratory Viruses, Paris, France; ^6^Alfort National Veterinary School, Maisons-Alfort, France

**Keywords:** Eastern Europe, next generation sequencing, ticks, quaranjaviruses, small ruminant

## Abstract

Ticks are involved in the transmission of various pathogens and several tick-borne diseases cause significant problems for the health of humans and livestock. The members of the *Quaranjavirus* genus are mainly associated with argas ticks but recent studies demonstrated the presence of novel quaranjaviruses-like in ixodid ticks. In 2020, 169 *Rhipicephalus sanguineus* ticks were collected in Southern Romania from small ruminants and analyzed by high-throughput transcriptome sequencing. Among the viral families that infect Romanian ticks, we have identified sequences from *Phenuiviridae* (Brown dog tick phlebovirus 1 [BDTPV1] and Brown dog tick phlebovirus 2 [BDTPV2]) and *Chuviridae* families (Cataloi mivirus [CTMV]), and numerous sequences from a new quaranjavirus-like, tentatively named Cataloi tick quaranjavirus (CTQV). Phylogenetic analyses performed on the five segments show that CTQV is phylogenetically positioned within a clade that encompasses *Ixodidae*-borne viruses associated with iguanas, small ruminants, seabirds, and penguins distributed across different geographical areas. Furthermore, CTQV is positioned differently depending on the segment considered. This is the first report on the detection of a quaranjavirus-like in Eastern Europe. Further investigations are needed to discern its infectivity and pathogenicity against vertebrates.

## Introduction

Ticks are major hematophagous arthropod vectors that harbor multiple pathogens for humans and animals. These pathogens are often vectored by specific tick species and therefore often restricted to geographical areas coinciding with the distribution of the arthropod. Such pathogens of medical importance include Crimean-Congo hemorrhagic fever virus (CCHFV) (1), Kyasanur Forest disease virus (KFDV) (2), tick-borne encephalitis virus (TBEV) (3), severe fever with thrombocytopenia syndrome virus (SFTSV) (3), Alkhurma virus (ALKV) (3, 4), and Heartland virus (HRTV) (5). Similarly, several tick-borne viruses also have threaten the health of livestock: these include the Africa swine fever virus (ASFV), the Nairobi sheep disease virus (NSDV), and the Louping ill virus (LIV), among others (6).

Moreover, the importation of animals from other areas and migratory birds enlarge the distribution of ticks, leading to exposure of new populations in remote areas (7). Romania is one of the most biogeographically diverse countries in Europe, with suitable conditions for the establishment of tick-borne virus foci, especially in the Southern half of the country that comprises the Danube Delta. Danube Delta constitutes a major hub for bird migration from Africa and Asia (8), leading to a high risk of the introduction of animal pathogens, such as zoonotic and vectored agents.

Despite previous reports that indicate that recently described tick-borne disease-causing agents (Tacheng tick virus 2, Jingmenvirus) and novel viruses that belong to viral families encompassing tick-borne arboviruses (Nayun tick nairovirus, Bole tick virus 4, and Sulina virus) circulate in the region (9, 10), the virome of ticks remains understudied in this area.

Taking advantage of the rapid development of next-generation sequencing (NGS) methods, many novel viral sequences have been identified in ticks of different species that are distributed in different regions of the world (11–17). For example, sequences that belong to new quaranjaviruses-like were identified in *Amblyomma dissimile* (18), removed from wild animals, *Ixodes uriae* (19) in the Arctic or in *Haemaphysalis hystricis* ticks that were collected in Japan (20). The quaranjaviruses belonging to this clade are distributed in various biotopes around the world, suggesting that there may still be many tick-borne orthomyxoviruses to be identified. Similarly, in the *Phenuiviridae* family, two novel phleboviruses named Brown dog tick phlebovirus 1 (BDTPV1) and Brown dog tick phlebovirus 2 (BDTPV2) (21) that present similarity in both the L and S segments to known phleboviruses but with an apparent lack of M segment coding for the glycoproteins (22) were detected. Since these viruses have so far been found only in ticks, their ability to infect vertebrates remains unknown. In complement to these viral families known to contain tick-borne viruses, novel families were recently identified. It is the case of the *Chuviridae* family, composed of ssRNA negative-strand viruses (23) associated with *Dermacentor* sp., *Haemaphysalis* sp. (24), and *Rhipicephalus sanguineus* or *Rhipicephalus microplus* ticks from French Antilles (25), Thailand (16), China, Brazil (26), and Trinidad and Tobago (18). To date, these miviruses have been predominantly identified in arthropods.

The aim of this work was to identify viruses associated with *R. sanguineus* ticks that were collected in Southern Romania from small ruminants, for a more comprehensive understanding of the viruses circulating in ticks and the potential risk for ruminants and humans living in the area.

## Materials and Methods

### Tick Collection

A total of 169 semi-engorged and engorged ticks were collected in Cataloi village (45°5′42″N 28°43′53″E), Tulcea County (Southern Romania) in October 2020 from the body-surface of adult sheep. This area represents a site located near the Danube Delta where ground-feeding migratory birds and small ruminants are abundant and in which agricultural practices and breeding could result in possible tick transfer from birds to mammals, such as humans. In short, living ticks from individual animals were placed into sterile tubes (all ticks removed from one animal in a single tube) and stored at 4 °C during transport. On arrival at the laboratory, ticks were morphologically identified at the genus level under a stereomicroscope using standard morphological key (27) and washed with ethanol 70% to remove external contaminants; each tick was cut lengthwise using sterile scalpels on an ice-cold surface Petri dish. Half was used for NGS analysis and the remainder was stored at −80°C for further virus isolation. To maintain the integrity of RNA, half-ticks to be sequenced were introduced in RNA later solution (Invitrogen, USA), according to best security practices of storing and transport. Samples were transferred to Institut Pasteur (Paris, France) for further processing.

### Nucleic Acid Extraction and Library Preparation

RNA extractions were conducted in a Biosafety Level 3 (BSL-3) laboratory. Ticks were pooled that resulted in a total of 14 pools (9 up to 16 ticks /pool). Total RNA was extracted from crushed materials using TRIzol Reagent (Invitrogen) and RNeasy mini kit (Qiagen) according to the recommendations of the manufacturers. The 14 pools of tick extracts were then combined to form one NGS library using NEBNext Ultra II DNA Library Prep Kit (New England Biolabs, EVRY, France) for Illumina, according to the manufacturer's instructions. NGS library was validated using an Agilent Bioanalyzer, quantified with a Qubit 2.0 Fluorometer (Invitrogen, USA) and sequencing was carried out on an Illumina NextSeq 500 Sequencer in a single-read 1 × 150 bp format to achieve approximately 65 million reads.

### Determination of Tick Species

To determine the species (and not only the genus) of ticks analyzed, we took advantage of the concomitant sequencing of tick transcriptome and used the Barcode of Life Data Systems (BOLD). Briefly, all trimmed reads were mapped onto the *Ixodidae* BOLD database, *de novo* assembled after extraction of mapped reads, and submitted to the BOLD Identification System. The identification was confirmed by BlastN. All ticks analyzed in this study were identified as *R. sanguineus*. In addition, to confirm these results, a nested PCR using cytochrome oxidase I (COI) universal primers was performed and sequenced by Sanger sequencing. Results confirmed the presence of *R. sanguineus* ticks in all the tick pools.

### Virus Assignation

As described previously (9), raw reads were processed with an in-house bioinformatics pipeline that comprises quality check and trimming, read normalization and assembly, open reading frames (ORFs) prediction, and taxonomic assignation of contigs and singletons using three successive specialized (RVDB-prot) (28) and generalist (NCBI/nr and NCBI/nt) databases.

### PCR Detection and Search for Endogenous Viral Elements (EVEs) of Relevant Viruses

The presence of viral RNA for CTQV, BDTPV1, BDTPV2, and Cataloi mivirus (CTMV) was confirmed by reverse transcription polymerase chain reaction (RT-PCR) for all the 14 tick pools using primers designed from the NGS sequences. Positive PCR products were purified with NucleoSpin Gel and PCR Clean-Up (Macherey-Nagel Germany) and sequenced by Sanger sequencing on the Eurofins Segenic Cologne platform. In order to explore the potential presence of integrated viral sequences into tick genomes for relevant viral species identified by NGS, tick samples were also screened by PCR by omitting the reverse transcription step.

## Results

The taxonomic assignation revealed that 17% of total sequences were assigned to viruses. Three viral families were identified: *Phenuiviridae* (41%), *Chuviridae* (36%), and *Orthomyxoviridae* (22%).

### *Phenuiviridae*- and *Chuviridae*-Related Viruses

The unique known viral genus among the *Phenuiviridae* family that is able to infect vertebrates, such as humans and domestic animals, is the *Phlebovirus* genus. The reads belonging to the *Phenuiviridae* family identified in our study were represented by BDTPV1 and BDTPV2, all negative sense bi-segmented single-stranded RNA (ssRNA) viruses that missed the M segment coding for the viral glycoprotein. The RNA-dependent RNA polymerase (RdRp) of Romanian BDTPV2 showed an amino-acid identity of 96.33% with its closest tick-borne Caribbean isolate relative (QDW81040 and QDW81041) while the nucleoprotein (NP) presented a lower amino-acid identity (93.88%). Romanian BDTPV2 presented a horizontal coverage of 99.57% for the L segment and 93.91% for the S segment (Table 1). Romanian BDTPV1 presented a horizontal coverage of 98.62% (L segment) and 93.87% (S segment) and amino-acid identity ranged from 96.79% (RdRp) to 92.55% (NP) with its closest relative BDTPV1 that is identified in Trinidad and Tobago (QDW81038 and QDW81039; Table 1).

**Table 1:** Viral sequences identified in ticks from South-Eastern Romania by high-throughput sequencing.

**Tick species**	**Order**	**Family**	**Genus**	**Best hit NCBI**	**Accesion number**	**Genome**	**Amino-acid %**
					**NCBI**	**coverage**	**identity**
*Rhipicephalus* sp.	*Bunyavirales*	*Phenuiviridae*	*Phlebovirus*	Brown dog tick phlevovirus 2	QDW81040	99.57%	96.33% (RdRp)
					QDW81041	93.91%	93.88% (NP)
				Brown dog tick phlevovirus 1	QDW81038	98.62%	96.79% (RdRp)
					QDW81039	93.87%	92.55% (NP)
	*Jingchuvirales*	*Chuivirdae*	*Mivirus*	Hebei mivirus 1	QYW06785	100%	94% (RdRp)
					QYW06786		93% (GP)
					QYW06787		83% (NP)
	*Articulavirales*	*Orthomyxoviridae*	*Quaranjavirus*	Granville quaranjavirus (HA/PA/PB1/PB2/NP)	UAJ23567	100%	64.8% (HA)
					UAJ23568	100%	66.7% (PA)
					UAJ23569	100%	70% (PB2)
					UAJ23565	57%	58% (NP)
				Zambezi tick virus 1	AWU49720	100%	89.3% (PB1)

**Figure 1:**
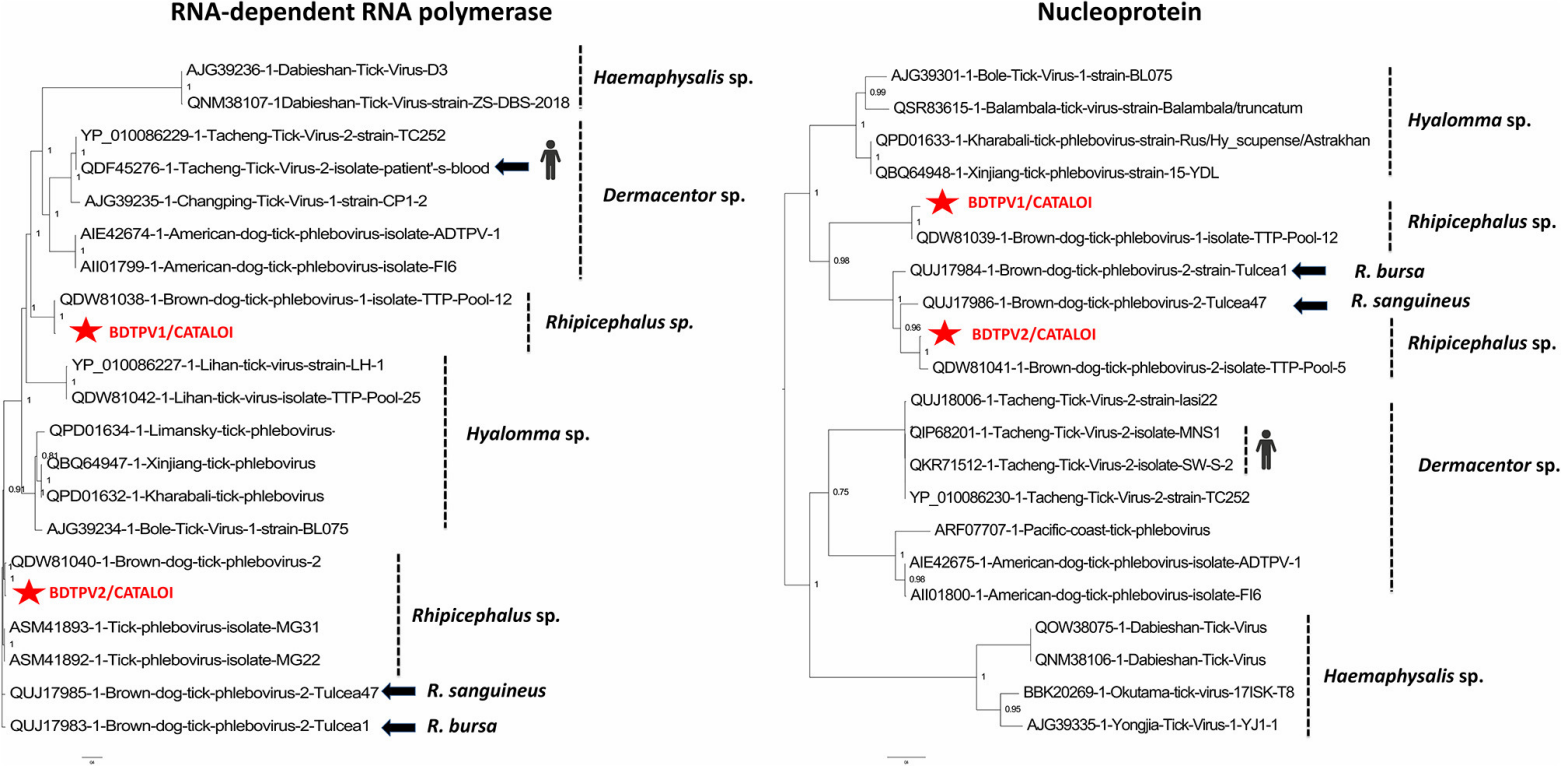
Phylogenetic relationship of nucleoprotein and RNA-dependent RNA polymerase of BDTPV1 and BDTPV2 identified in Romanian *Rhipicephalus* ticks with other viruses among the *Phlebovirus* genus.

Phylogenetic analysis performed on the amino-acid sequences placed Romanian phleboviruses within the *Uukuvirus* genus. Romanian BDTPV2 was clustered in a clade encompassing tick-borne isolates from Trinidad and Tobago. Romanian BDTPV2 was clustered in a highly supported distinct clade (posterior probability of 1), different from other strains also originating from Southern Romania (the same region as in the present study), suggesting the presence of two different strains of BDTPV2 in the same area and a possible geographical and/or host specificity of these viruses. Romanian BDTPV2 was placed within a clade apparently restricted to tick phleboviruses that were primarily identified in *Rhipicephalus* (*bursa* or *sanguineus*) ticks (Figure 1). Phylogenetic analysis placed Romanian BDTPV1 in a clade that includes a tick-borne Caribbean isolate identified in *R. sanguineus*, showing a possible host specificity for this virus (Figure 1). The presence of viral RNA for BDTPV1 and BDTPV2 was confirmed by RT-PCR and all tick pools screened for the presence of EVEs were negative confirming that these sequences were not endogenous to the ticks.

**Figure 2:**
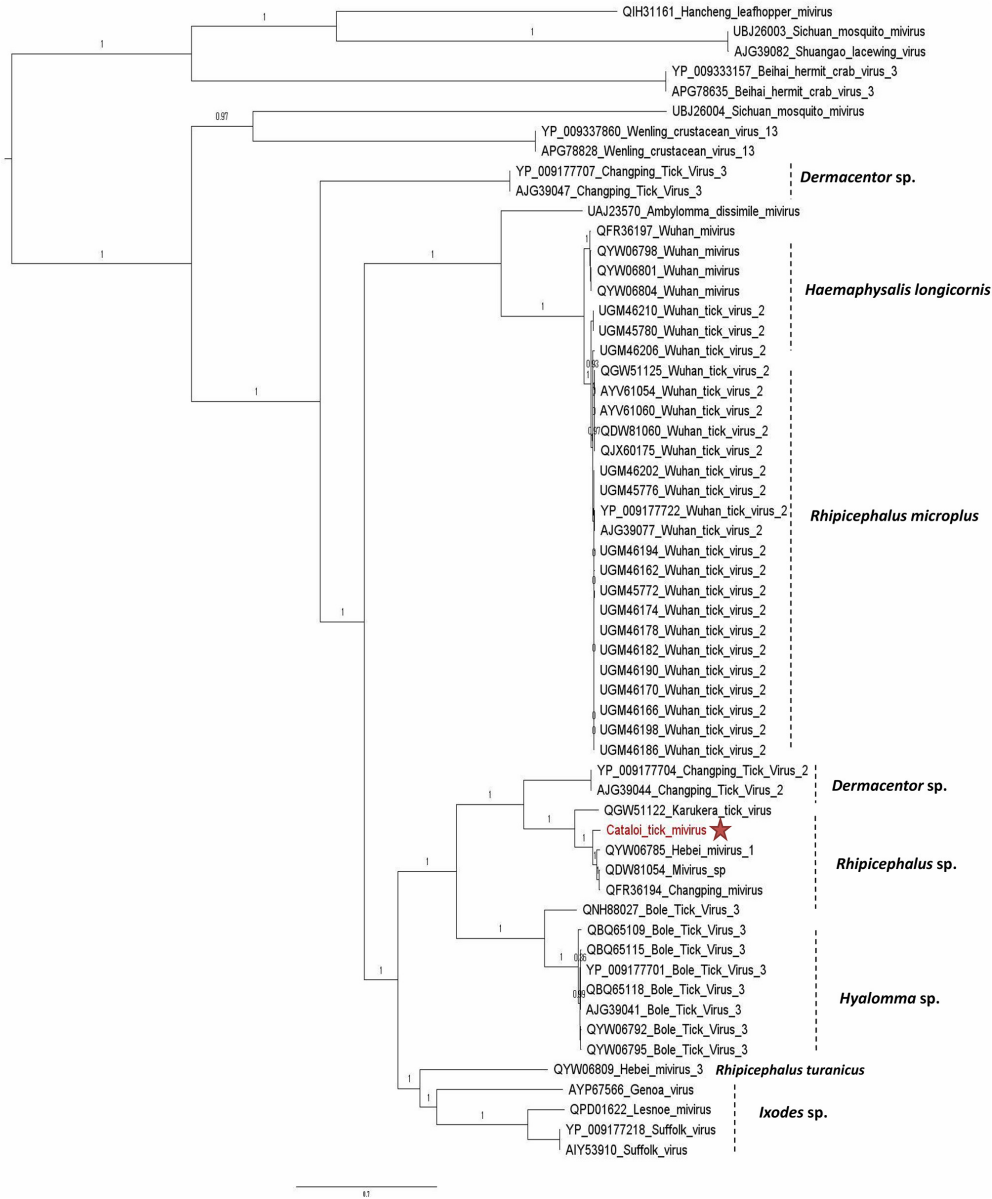
Phylogenetic relationship of RNA-dependent RNA polymerase of Cataloi mivirus (CTMV) identified in Romanian *Rhipicephalus* ticks with other viruses among the *Chuviridae* family.

Sequences that belong to *Chuviridae* family were also identified. *Chuviridae* is a recently recognized viral family among the *Jingchuvirales* that was primarily assigned to the *Mononegavirales* order, infecting a wide variety of invertebrate hosts, i.e., Crustacea, Nematoda, Insecta, Myriapoda, Arachnida, and Ixodida. Both *Argasidae* (Argas mivirus) and *Ixodidae* ticks are infected by tick-borne chuviruses (e.g., Brown dog tick mivirus, Bole mivirus, Changping mivirus, Lonestar mivirus, Suffolk mivirus, and Wuhan mivirus). The complete genome of CTMV identified in the present study comprised three ORFs: the first ORF codes for a large protein of 2,156 amino-acid corresponding to the RdRp of the virus; ORF2 codes for the putative glycoprotein of 683 amino-acid, and ORF3 codes for the NP of 527 amino-acid. CTMV showed an amino-acid identity ranging from 83% for the NP to 94% for the polymerase with its closest tick-borne Chinese isolate identified in *R. turanicus* (QYW06785, QYW06786, and QYW06787; Table 1) and a genome coverage of 100%. Phylogenetic analysis performed on the polymerase amino-acid sequences placed CTMV in a specific clade comprising *Rhipicephalus* sp. ticks isolates originating from China, Trinidad and Tobago, and Thailand, suggesting that these miviruses may present a possible specialization to their tick vector (Figure 2). The presence of viral RNA for CTMV was confirmed by RT-PCR and all tick pools screened for the presence of EVE were negative, confirming that these sequences were not endogenous to the ticks.

### *Orthomyxoviridae* Family

The *Orthomyxoviridae* family is composed of seven genera: types A, B, C, and D *Influenza virus, Isavirus, Thogotovirus*, and *Quaranjavirus*. Viruses in this family infect a broad range of hosts, such as humans, birds, swine, fish, and arthropods (29).

As results of RNA virome analysis, 633,626 reads (22% of all viral reads) were classified within the *Orthomyxoviridae* family. Sequences of a novel quaranjavirus, tentatively named CTQV, were identified in engorged *Rhipicephalus* sp. ticks that were collected on small ruminants, in Southern Romania. CTQV sequences comprise five segments with homology to known quaranjaviruses: the NP, the hemagglutinin (HA), and three polymerase subunits (PB1, PB2, and PA).

Within the HA, PA, PB2, and NP segments, CTQV was most similar to Granville quaranjavirus (GQV), a novel quaranjavirus identified in *A. dissimile* ticks removed from iguanas, from Trinidad and Tobago (UAJ23567, UAJ23568, UAJ23569, and UAJ23565). CTQV presented a horizontal genome coverage ranging from 57 to 100%, depending on the segment (Table 1). CTQV differed from its Caribbean counterpart by presenting between 58 and 70% amino-acid identity, depending on the protein considered, indicating that the virus detected in this study is probably a new member of the *Quaranjavirus* genus or a related group. In contrast with other segments that were similar to GQV, PB1 protein (considered the most conserved of the orthomyxovirus genes) shared 89% amino-acids identity to Zambezi tick virus 1 (ZaTV-1), a highly divergent quaranjavirus identified in *Rhipicephalus* ticks from Mozambique (AWU49720). Unfortunately, additional segments for ZaTV-1 were not available for analysis. The presence of viral RNA for CTQV was confirmed by RT-PCR and all tick pools screened for the presence of EVE were negative.

**Figure 3:**
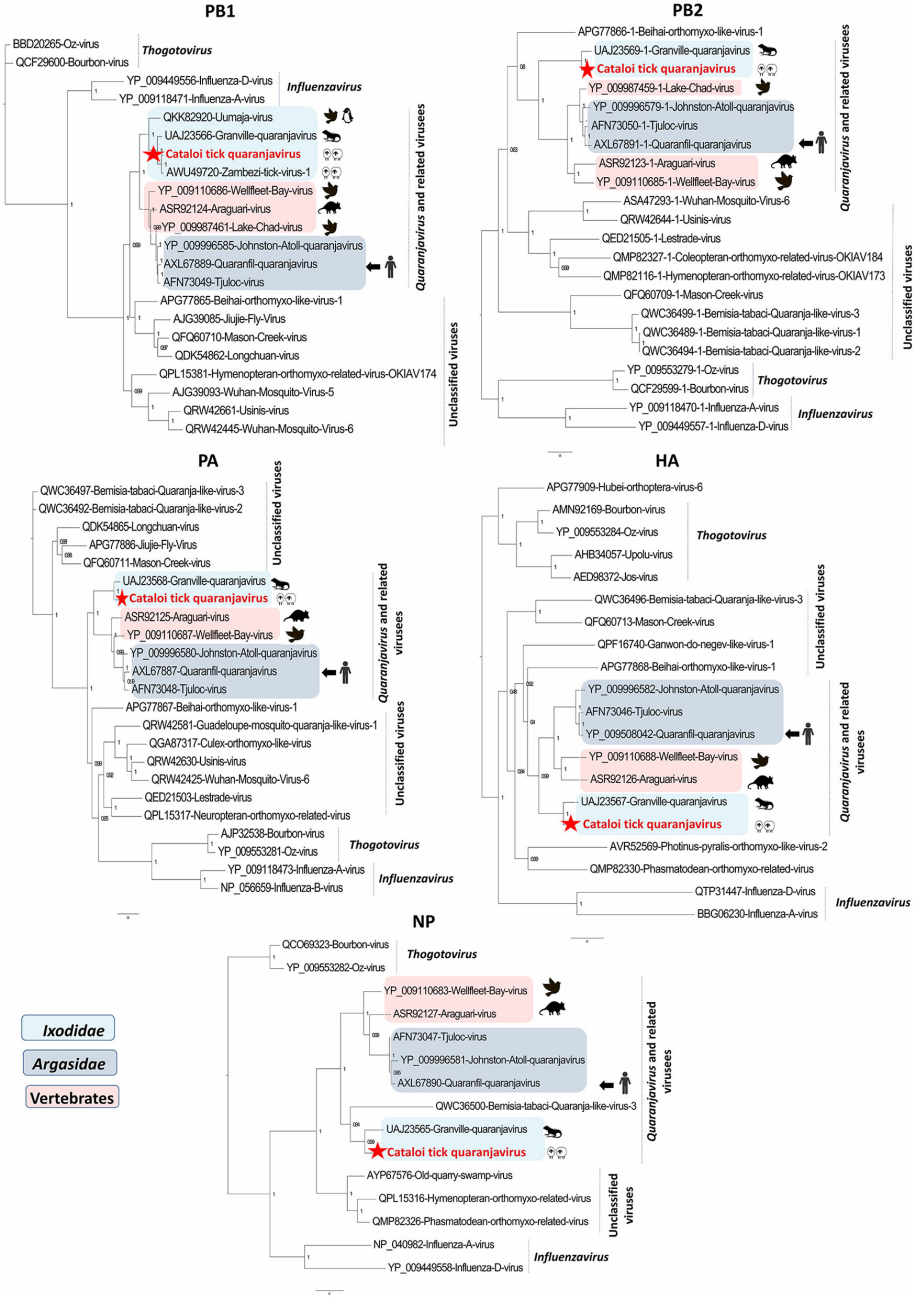
Phylogenetic analysis of Cataloi tick quaranjavirus (CTQV), PB1, PB2, PA, NP, and HA proteins with other viruses in the *Orthomyxoviridae* family. In light blue: *Ixodidae*-associated quaranjaviruses; in dark blue: *Argasidae*-associated quaranjaviruses, in pink: vertebrates associated quaranjaviruses.

Phylogenetic analyses performed on the five segments show that CTQV that is positioned differently depends on the segment. Generally, CTQV was clustered with quaranjaviruses and related viruses, which are distributed across different geographical areas. Within the PA, PB2, HA, and NP proteins, CTQV formed a separate branch, being placed in a clade with other recently tick-borne quaranjaviruses identified in ixodid ticks from Caribe (Granville virus) and Japan (Ohshima virus) while CTQV was phylogenetically close to ZaTV-1 in PB1 protein (Figure 3 and Supplementary Figure S1). Unfortunately, isolation attempts of CTQV that were performed on VeroE6 cells and embryonated chicken eggs inoculated with CTQV positive pools of ticks were unsuccessful. The presence of each viral segment of CTQV was confirmed by RT-PCR. However, these segments could derive from multiple viral strains that were carried by different individual ticks, given the fact that ticks were pooled before extraction. All tick pools screened for the presence of EVE were negative.

## Discussion

In this study, we performed a viral meta-transcriptomic analysis to identify viruses that are associated with *Ixodidae* ticks, collected in the Southern half of Romania that comprised the Danube Delta Biosphere Reserve (DDBR), the second largest wetland in Europe. Despite the fact that DDBR is a very rich area in terms of diversity, the surveillance of tick-associated viruses is limited. Identifying a list of viruses at higher risk of emergence requires first the characterization of the ecological cycle of the virus that includes its putative reservoir hosts, hematophagous arthropod vectors (in the case of arboviruses), and vertebrate recipient hosts (30). There are few studies regarding the presence of quaranjaviruses in hard ticks worldwide and little is known about the molecular pathogenesis of these viruses.

To our knowledge, this is the first report on the detection of a quaranjavirus-like virus that is associated with ixodid ticks in the Eastern Europe region. *Quaranjavirus* genus (*Orthomyxoviridae* family) is a group of negative-sense segmented viruses, with a single-stranded genome and which generally is divided into six segments (polymerase acidic protein-PA, polymerase basic protein 1-PB1, polymerase basic protein 2-PB2, hemagglutinin protein-HA, nucleocapsid protein-NP, and matrix protein-M). The genus *Quaranjavirus* contains two virus species recognized by International Committee on Taxonomy of Viruses (ICTV): Johnston Atoll (JAV) virus and Quaranfil virus (QRFV), known to be transmitted to birds mainly by argasid ticks. Several strains of QRFV have been isolated from ticks and seabirds in Egypt (31), South Africa (32), Afghanistan, Nigeria (33), Kuwait, Iraq, Yemen (34), and Iran (35). Quaranfil quaranjavirus is the only member of the genus that has been shown to infect humans. It was isolated from soft ticks and from the blood of children with mild febrile illness in Quaranfil, Egypt (36). Furthermore, additional quaranjavirus-like viruses have been isolated from argasid ticks (37, 38) and their vertebrate hosts (Araguari virus from mammals and Wellfleet Bay virus from birds) (39, 40).

More recent studies have also identified quaranjavirus-like sequences in hard ticks, for example, sequences of a novel quaranjavirus, tentatively named GQV, were identified in *A. dissimile* ticks that were removed from iguanas (18). Related viruses have been reported in *Rhipicephalus* sp. ticks from Mozambique (41), in *H. hystricis* from Japan (20), and in *I. uriae* ticks from Sweden (19). Since these viruses have so far been found only in ticks, their ability to infect vertebrates remains unknown.

In our study, CTQV was found in engorged *Rhipicephalus* sp., a three-host tick that can infest a wide range of domestic and wild hosts, such as cats, rodents, birds, cattle, horses, goats, and humans (42–44). CTQV is phylogenetically positioned within a clade apparently restricted to *Ixodidae* and distinct from argas-related quaranjaviruses. This apparent high diversity of vertebrate hosts (iguanas, small ruminants, and seabirds) suggests that CTQV and other *Ixodidae*-associated quaranjaviruses probably harbor a tick specificity, independently of the hosts on which ticks feed (Supplementary Figure S2). The ability of ixodid-associated quaranjaviruses to replicate in their vertebrate hosts remains to be elucidated. Similarly, CTQV detected in Eastern Romania was clustered in a clade with other viruses that were originated from different geographical biotopes, such as Trinidad and Tobago, Mozambique, and Sweden, indicating that no geographical specificity shapes the evolution history of these viruses and reinforcing the hypothesis about the vector specificity of these viruses.

Viruses in the *Orthomyxoviridae* family have the genetic ability to undergo reassortment of the genome segments within a given genus. Phylogenetic analysis performed on the five segments showed that CTQV was positioned differently depending on the segment, suggesting a possible reassortment at the origin of CTQV. However, the lack of a complete genome for ZaTV-1 may result in a biased reassortment analysis for CTQV.

To conclude, we performed a viral meta-transcriptomic analysis of *R. sanguineus* ticks that were collected in the Danube Delta region from Southern Romania to identify known or novel viruses. We detected viral sequences phylogenetically close to known *Phenuiviridae* and *Chuviridae* members (BDTP 1 and 2, and Hebei mivirus 1, respectively). In addition, a novel quaranjavirus-like restricted to hard ticks and distinct from argas-related quaranjaviruses was identified. This study constitutes the first step toward a more comprehensive overview of the quaranjaviruses diversity in ixodid ticks and increases the knowledge of the diversity of viruses carried by ticks from Eastern Europe. Future studies are required to establish the pathogenic potential of CTQV and other viruses for animals and humans.

## Data Availability Statement

The datasets presented in this study can be found in online repositories. The names of the repository/repositories and accession number(s) can be found below: https://www.ncbi.nlm.nih.gov/genbank/, OM405131; https://www.ncbi.nlm.nih.gov/genbank/, OM405132; https://www.ncbi.nlm.nih.gov/genbank/, OM405133; https://www.ncbi.nlm.nih.gov/genbank/, OM405134; https://www.ncbi.nlm.nih.gov/genbank/, OM405135; https://www.ncbi.nlm.nih.gov/genbank/, OM405136; https://www.ncbi.nlm.nih.gov/genbank/, OM405137; https://www.ncbi.nlm.nih.gov/genbank/, OM405138; https://www.ncbi.nlm.nih.gov/genbank/, OM405139; https://www.ncbi.nlm.nih.gov/genbank/, OM405140.

## Author Contributions

BB: conceptualization, formal analysis, resources, investigation, writing-original draft, and visualization. ST: conceptualization, investigation, writing-reviewing, and editing. SM: investigation and resources. DC: investigation and formal analysis. TB: data analysis. SW and GS: supervision and reviewing. ME: conceptualization, supervision, reviewing, and editing. All authors read and approved the final manuscript.

## Funding

This study was support by Institut Pasteur, Labex IBEID (ANR-10-LABX-62-IBEID).

## Conflict of Interest

The authors declare that the research was conducted in the absence of any commercial or financial relationships that could be construed as a potential conflict of interest.

## Publisher's Note

All claims expressed in this article are solely those of the authors and do not necessarily represent those of their affiliated organizations, or those of the publisher, the editors and the reviewers. Any product that may be evaluated in this article, or claim that may be made by its manufacturer, is not guaranteed or endorsed by the publisher.

## References

[1] Bente DA Forrester NL Watts DM McAuley AJ Whitehouse CA Bray M. Crimean-Congo hemorrhagic fever: history, epidemiology, pathogenesis, clinical syndrome and genetic diversity. Antiviral Res. (2013) 100:159–89. 10.1016/j.antiviral.2013.07.006 23906741

[2] Holbrook MR. Kyasanur forest disease. Antiviral Res. (2012) 96:353–62. 10.1016/j.antiviral.2012.10.005 23110991 PMC3513490

[3] Yu XJ Liang MF Zhang SY Liu Y Li JD Sun YL. Fever with thrombocytopenia associated with a novel bunyavirus in China. N Engl J Med. (2011) 364:1523–32. 10.1056/NEJMoa1010095 21410387 PMC3113718

[4] Labuda M Nuttall PA. Tick-borne viruses. Parasitology. (2004) 129(Suppl.):S221–45. 10.1017/S0031182004005220 15938513

[5] McMullan LK Folk SM Kelly AJ MacNeil A Goldsmith CS Metcalfe MG. A new phlebovirus associated with severe febrile illness in Missouri. N Engl J Med. (2012) 367:834–41. 10.1056/NEJMoa1203378 22931317

[6] Dantas-Torres F Chomel BB Otranto D. Ticks and tick-borne diseases: a One Health perspective. Trends Parasitol. (2012) 28:437–46. 10.1016/j.pt.2012.07.003 22902521

[7] Ogden NH Mechai S Margos G. Changing geographic ranges of ticks and tick-borne pathogens: drivers, mechanisms and consequences for pathogen diversity. Front Cell Infect Microbiol. (2013) 3:46. 10.3389/fcimb.2013.00046 24010124 PMC3756306

[8] Hanganu J Dubyna D Zhmud E Grigoraş I Menke U Drost H. Vegetation of the Biosphere Reserve “Danube Delta". Tulcea: Danube Delta National Institute (2002).

[9] Bratuleanu BE Temmam S Chretien D Regnault B Perot P Bouchier C. The virome of rhipicephalus, dermacentor and haemaphysalis ticks from eastern romania includes novel viruses with potential relevance for public health. Transbound Emerg Dis. (2021) 1–17. 10.1111/tbed.14105 33840161

[10] Tomazatos A von Possel R Pekarek N Holm T Rieger T Baum H. Discovery and genetic characterization of a novel orthonairovirus in Ixodes ricinus ticks from Danube Delta. Infect Genet Evol. (2021) 88:104704. 10.1016/j.meegid.2021.104704 33418146

[11] Tokarz R Sameroff S Leon MS Jain K Lipkin WI. Genome characterization of Long Island tick rhabdovirus, a new virus identified in Amblyomma americanum ticks. Virol J. (2014) 11:26. 10.1186/1743-422X-11-26 24517260 PMC3928085

[12] Xia H Hu C Zhang D Tang S Zhang Z Kou Z. Metagenomic profile of the viral communities in Rhipicephalus spp. ticks from Yunnan, China PLoS ONE. (2015) 10:e0121609. 10.1371/journal.pone.0121609 25799057 PMC4370414

[13] Shi M Lin XD Tian JH Chen LJ Chen X Li CX. Redefining the invertebrate RNA virosphere. Nature. (2016) 540:539–43. 10.1038/nature20167 27880757

[14] Pettersson JH Shi M Bohlin J Eldholm V Brynildsrud OB Paulsen KM. Characterizing the virome of Ixodes ricinus ticks from northern Europe. Sci Rep. (2017) 7:10870. 10.1038/s41598-017-11439-y 28883464 PMC5589870

[15] Temmam S Bigot T Chretien D Gondard M Perot P Pommelet V. Insights into the host range, genetic diversity, and geographical distribution of jingmenviruses. mSphere. (2019) 4:1–13. 10.1128/mSphere.00645-19 31694898 PMC6835211

[16] Temmam S Chretien D Bigot T Dufour E Petres S Desquesnes M. Monitoring silent spillovers before emergence: a pilot study at the tick/human interface in thailand. Front Microbiol. (2019) 10:2315. 10.3389/fmicb.2019.02315 31681195 PMC6812269

[17] Wille M Harvey E Shi M Gonzalez-Acuna D Holmes EC Hurt AC. Sustained RNA virome diversity in Antarctic penguins and their ticks. ISME J. (2020) 14:1768–82. 10.1038/s41396-020-0643-1 32286545 PMC7305176

[18] Sameroff S Tokarz R Jain K Oleynik A Carrington CVF Lipkin WI. Novel quaranjavirus and other viral sequences identified from ticks parasitizing hunted wildlife in Trinidad and Tobago. Ticks Tick Borne Dis. (2021) 12:101730. 10.1016/j.ttbdis.2021.101730 33957484

[19] Pettersson JH Ellstrom P Ling J Nilsson I Bergstrom S Gonzalez-Acuna D. Circumpolar diversification of the Ixodes uriae tick virome. PLoS Pathog. (2020) 16:e1008759. 10.1371/journal.ppat.1008759 32745135 PMC7425989

[20] Kobayashi D Kuwata R Kimura T Faizah AN Higa Y Hayashi T. Detection of quaranjavirus-like sequences from Haemaphysalis hystricis ticks collected in Japan. Jpn J Infect Dis. (2021) 75:195–8. 10.7883/yoken.JJID.2021.129 34470960

[21] Sameroff S Tokarz R Charles RA Jain K Oleynik A Che X. Viral Diversity of tick species parasitizing cattle and dogs in trinidad and tobago. Sci Rep. (2019) 9:10421. 10.1038/s41598-019-46914-1 31320705 PMC6639388

[22] Abudurexiti A Adkins S Alioto D Alkhovsky SV Avsic-Zupanc T Ballinger MJ. Taxonomy of the order Bunyavirales: update 2019. Arch Virol. (2019) 164:1949–65. 10.1007/s00705-019-04253-6 31065850 PMC6641860

[23] Siddell SG Walker PJ Lefkowitz EJ Mushegian AR Adams MJ Dutilh BE. Additional changes to taxonomy ratified in a special vote by the International Committee on Taxonomy of Viruses (October 2018). Arch Virol. (2019) 164:943–6. 10.1007/s00705-018-04136-2 30663020

[24] Brinkmann A Dincer E Polat C Hekimoglu O Hacioglu S Foldes K. A metagenomic survey identifies Tamdy orthonairovirus as well as divergent phlebo-, rhabdo-, chu- and flavi-like viruses in Anatolia, Turkey. Ticks Tick Borne Dis. (2018) 9:1173–83. 10.1016/j.ttbdis.2018.04.017 29728337

[25] Gondard M Temmam S Devillers E Pinarello V Bigot T Chretien D. RNA viruses of amblyomma variegatum and rhipicephalus microplus and cattle susceptibility in the French Antilles. Viruses. (2020) 12:144. 10.3390/v12020144 31991915 PMC7077237

[26] Souza WM Fumagalli MJ Torres Carrasco AO Romeiro MF Modha S Seki MC. Viral diversity of Rhipicephalus microplus parasitizing cattle in southern Brazil. Sci Rep. (2018) 8:16315. 10.1038/s41598-018-34630-1 30397237 PMC6218518

[27] Perez-Eid C. Les tiques. Identification, biologie, importance medicale et veterinaire. Paris: Tec & Doc Lavoisier (2007).

[28] Bigot T Temmam S Perot P Eloit M. RVDB-prot, a reference viral protein database and its HMM profiles. F1000Res. (2019) 8:530. 10.12688/f1000research.18776.1 32983411 PMC7492780

[29] Webster RG Bean WJ Gorman OT Chambers TM Kawaoka Y. Evolution and ecology of influenza A viruses. Microbiol Rev. (1992) 56:152–79. 10.1128/mr.56.1.152-179.1992 1579108 PMC372859

[30] Temmam S Davoust B Berenger JM Raoult D Desnues C. Viral metagenomics on animals as a tool for the detection of zoonoses prior to human infection? Int J Mol Sci. (2014) 15:10377–97. 10.3390/ijms150610377 24918293 PMC4100157

[31] Al-Khalifa MS Diab FM Khalil GM. Man-threatening viruses isolated from ticks in Saudi Arabia. Saudi Med J. (2007) 28:1864–7. 18060218

[32] Sang R Onyango C Gachoya J Mabinda E Konongoi S Ofula V. Tickborne arbovirus surveillance in market livestock, Nairobi, Kenya. Emerg Infect Dis. (2006) 12:1074–80. 10.3201/eid1207.060253 16836823 PMC3291068

[33] Kemp GE Lee VH Moore DL. Isolation of Nyamanini and Quaranfil viruses from Argas (Persicargas) arboreus ticks in Nigeria. J Med Entomol. (1975) 12:535–7. 10.1093/jmedent/12.5.535 1223303

[34] Converse JD Moussa MI. Quaranfil virus from Hyalomma dromedarii (Acari: Ixodoidea) collected in Kuwait, Iraq and Yemen. J Med Entomol. (1982) 19:209–10. 10.1093/jmedent/19.2.209 7086857

[35] Sureau P Klein JM. [Arboviruses in Iran (author's transl)]. Med Trop (Mars). (1980) 40:549–54. 7442513

[36] Taylor RM Hurlbut HS Work TH Kingston JR Hoogstraal H. Arboviruses isolated from ARGAS TICKS IN Egypt: Quaranfil, Chenuda, and Nyamanini. Am J Trop Med Hyg. (1966) 15:76–86. 10.4269/ajtmh.1966.15.76 5901633

[37] Presti RM Zhao G Beatty WL Mihindukulasuriya KA da Rosa AP Popov VL. Quaranfil, Johnston Atoll, and Lake Chad viruses are novel members of the family Orthomyxoviridae. J Virol. (2009) 83:11599–606. 10.1128/JVI.00677-09 19726499 PMC2772707

[38] Mourya DT Yadav PD Nyayanit DA Majumdar TD Jain S Sarkale P. Characterization of a strain of quaranfil virus isolated from soft ticks in India. Is quaranfil virus an unrecognized cause of disease in human and animals?” Heliyon. (2019) 5:e01368. 10.1016/j.heliyon.2019.e01368 30957047 PMC6431747

[39] Da Silva EV Da Rosa AP Nunes MR Diniz JA Tesh RB Cruz AC. Araguari virus, a new member of the family Orthomyxoviridae: serologic, ultrastructural, and molecular characterization. Am J Trop Med Hyg. (2005) 73:1050–8. 10.4269/ajtmh.2005.73.1050 16354811

[40] Allison AB Ballard JR Tesh RB Brown JD Ruder MG Keel MK. Cyclic avian mass mortality in the northeastern United States is associated with a novel orthomyxovirus. J Virol. (2015) 89:1389–403. 10.1128/JVI.02019-14 25392223 PMC4300652

[41] Cholleti H Hayer J Mulandane FC Falk K Fafetine J Berg M. Viral metagenomics reveals the presence of highly divergent quaranjavirus in Rhipicephalus ticks from Mozambique. Infect Ecol Epidemiol. (2018) 8:1478585. 10.1080/20008686.2018.1478585 29868166 PMC5974704

[42] Estrada-Pena A Jongejan F. Ticks feeding on humans: a review of records on human-biting Ixodoidea with special reference to pathogen transmission. Exp Appl Acarol. (1999) 23:685–715. 10.1023/A:1006241108739 10581710

[43] Walker JB Keirans JE Horak I. The genus Rhipicephalus (Acardi, Ixodidae): A Guide to the Brown Ticks of the World. Cambridge; New York, NY: Cambridge University Press (2000).

[44] Dantas-Torres F Ferreira DR de Melo LM Lima PA Siqueira DB Rameh-de-Albuquerque LC. Ticks on captive and free-living wild animals in northeastern Brazil. Exp Appl Acarol. (2010) 50:181–9. 10.1007/s10493-009-9296-5 19693679

