# Supplementary material for: Detection of Phenuiviridae, Chuviridae Members, and a Novel Quaranjavirus in Hard Ticks From Danube Delta
Source: Front Vet Sci. 2022 Apr 13;9:863814. doi: 10.3389/fvets.2022.863814 (PMC9044029; doi:10.3389/fvets.2022.863814)
Supplement: Supplementary file 1 [file Table_1.DOCX]

Supplementary Material

# Supplementary Figures


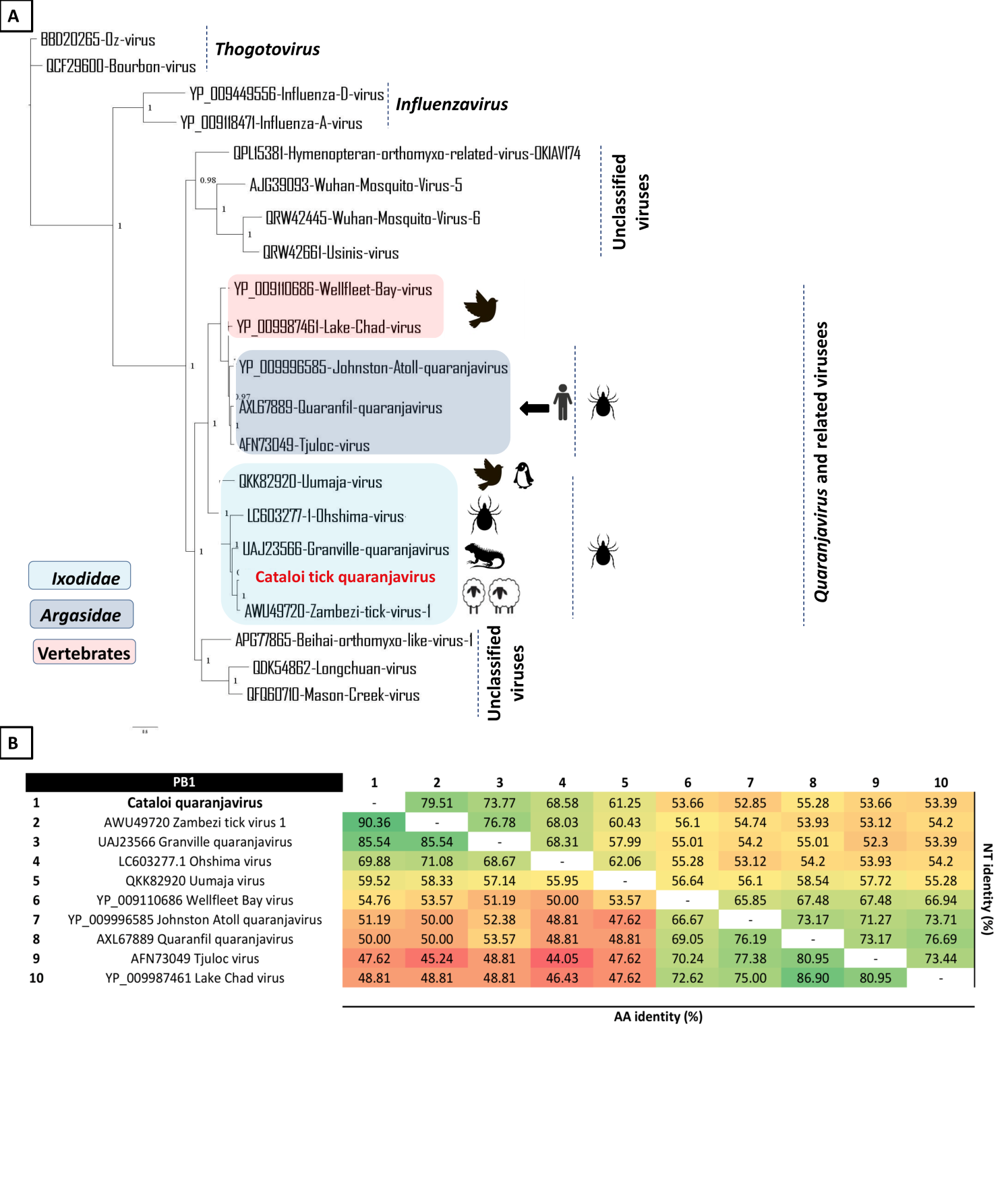


**Supplementary Figure 1.** **A.** Partial phylogenetic reconstruction of Romanian *Rhipicephalus* CTQV and other *Orthomyxoviridae* viruses based on PB1 protein. In light blue: *Ixodidae*- associated quaranjaviruses; in dark blue: *Argasidae*-associated quaranjaviruses, in pink: vertebrates associated quaranjaviruses. **B.** Amino-acid (lower) and nucleotide (upper) identity matrices of Cataloi tick quaranjavirus and other *Quaranjavirus*-related viruses. Sequences were aligned with MAFFT, and identity matrices were constructed using CLC Main Workbench 21.0.4 (Qiagen). Matrices were colored according to the identity scale, from 44% (red) to 91% (green) of nucleotide or amino-acid identity.


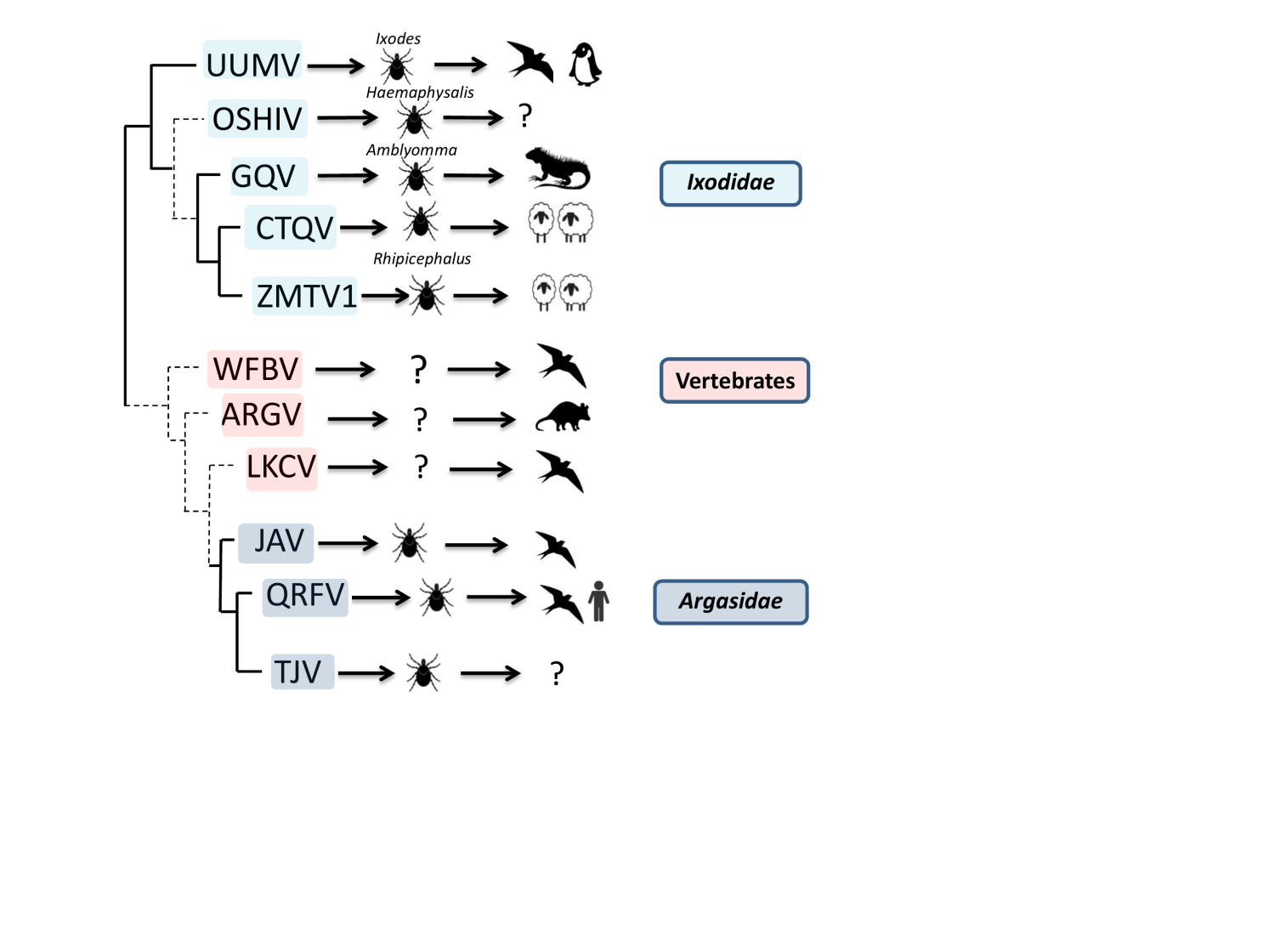


**Supplementary Figure 2.** Schematic representation of *Quaranjavirus* relationship with their corresponding vectors and associated vertebrate hosts**.** Continuous lines represent constant phylogenetic clustering across the different segments; dashed lines correspond to different phylogenetic positioning among the segments.
